# Metabolic syndrome influences cardiac gene expression pattern at the transcript level in male ZDF rats

**DOI:** 10.1186/1475-2840-12-16

**Published:** 2013-01-15

**Authors:** Márta Sárközy, Ágnes Zvara, Nóra Gyémánt, Veronika Fekete, Gabriella F Kocsis, Judit Pipis, Gergő Szűcs, Csaba Csonka, László G Puskás, Péter Ferdinandy, Tamás Csont

**Affiliations:** 1 Cardiovascular Research Group, Department of Biochemistry, Faculty of Medicine, University of Szeged, Szeged, Hungary; 2 Department of Functional Genomics, Biological Research Center, Szeged, Hungary; 3 Pharmahungary Group, Szeged, Hungary; 4 Department of Pharmacology and Pharmacotherapy, Faculty of Medicine, Semmelweis University, Budapest, Hungary

**Keywords:** Metabolic syndrome, Diabetes mellitus type 2, Hyperlipidemia, Myocardium, DNA microarray, Heart, GO analysis

## Abstract

**Background:**

Metabolic syndrome (coexisting visceral obesity, dyslipidemia, hyperglycemia, and hypertension) is a prominent risk factor for cardiovascular morbidity and mortality, however, its effect on cardiac gene expression pattern is unclear. Therefore, we examined the possible alterations in cardiac gene expression pattern in male Zucker Diabetic Fatty (ZDF) rats, a model of metabolic syndrome.

**Methods:**

Fasting blood glucose, serum insulin, cholesterol and triglyceride levels were measured at 6, 16, and 25 wk of age in male ZDF and lean control rats. Oral glucose tolerance test was performed at 16 and 25 wk of age. At week 25, total RNA was isolated from the myocardium and assayed by rat oligonucleotide microarray for 14921 genes. Expression of selected genes was confirmed by qRT-PCR.

**Results:**

Fasting blood glucose, serum insulin, cholesterol and triglyceride levels were significantly increased, glucose tolerance and insulin sensitivity were impaired in ZDF rats compared to leans. In hearts of ZDF rats, 36 genes showed significant up-regulation and 49 genes showed down-regulation as compared to lean controls. Genes with significantly altered expression in the heart due to metabolic syndrome includes functional clusters of metabolism (e.g. 3-hydroxy-3-methylglutaryl-Coenzyme A synthase 2; argininosuccinate synthetase; 2-amino-3-ketobutyrate-coenzyme A ligase), structural proteins (e.g. myosin IXA; aggrecan1), signal transduction (e.g. activating transcription factor 3; phospholipase A2; insulin responsive sequence DNA binding protein-1) stress response (e.g. heat shock 70kD protein 1A; heat shock protein 60; glutathione S-transferase Yc2 subunit), ion channels and receptors (e.g. ATPase, (Na^+^)/K^+^ transporting, beta 4 polypeptide; ATPase, H^+^/K^+^ transporting, nongastric, alpha polypeptide). Moreover some other genes with no definite functional clusters were also changed such as e.g. S100 calcium binding protein A3; ubiquitin carboxy-terminal hydrolase L1; interleukin 18. Gene ontology analysis revealed several significantly enriched functional inter-relationships between genes influenced by metabolic syndrome.

**Conclusions:**

Metabolic syndrome significantly alters cardiac gene expression profile which may be involved in development of cardiac pathologies in the presence of metabolic syndrome.

## Introduction

It is well established that metabolic syndrome is a major risk factor for cardiovascular diseases [1-4]. Metabolic syndrome is defined as the coexistence of visceral obesity, dyslipidemia, hyperglycemia, and hypertension [5,6]. Most individuals with metabolic syndrome have abdominal obesity and develop insulin resistance, therefore the prevalence of metabolic syndrome and pre-diabetes overlap [7,8]. In addition, metabolic syndrome can be considered as a direct precursor state of diabetes mellitus type 2 [7,9] and cardiovascular diseases [7,10]. Moreover, the efficacy of cardioprotective interventions (i.e. pre- and postconditioning) seems to be diminished in the presence of pathological conditions associated with metabolic syndrome [11-13] such as obesity [14], diabetes [15-18] or dyslipidemia [19,20]. Metabolic syndrome affects a large population including all ages from children to elderly and both sexes worldwide [21-23]. According to the Third National Health and Nutrition Examination Survey (NHANES III) criteria, about 47 million people (approximately 24% of the US adult population) had metabolic syndrome in the USA in 2002 [24]. Its prevalence is raising both in developed [21,24], and in developing countries [21,24]. In addition, patients suffering from metabolic syndrome have an approximate 5-fold increase in diabetes risk compared with persons without metabolic syndrome [7,25]. The effect of metabolic syndrome on gene expression pattern in various tissue types has been investigated in a few studies. In insulin sensitive tissues (liver, skeletal muscle and adipose tissue) [26] and pancreatic β-cells [27] obtained from the well-known metabolic syndrome model (Zucker Diabetic Fatty rat, ZDF), altered gene expression pattern were shown when compared to their controls. However, the effect of metabolic syndrome on the gene expression pattern of the heart has not been investigated yet.

Therefore, our aim was in the present study to investigate the effect of metabolic syndrome on cardiac gene expression pattern in male ZDF rats.

## Materials and methods

This investigation conforms to the National Institutes of Health Guide for the Care and Use of Laboratory Animals (NIH Pub. No. 85-23, Revised 1996) and was approved by the Animal Research Ethics Committee of the University of Szeged.

Male Zucker Diabetic Fatty (ZDF/Gmi-fa/fa) rats and their lean controls were obtained from Charles River Laboratories at the age of 5 weeks and were housed at 22±2°C with a 12:12-h light-dark cycle. The rats received Purina 5008 chow and water ad libitum for 20 weeks after their arrival.

The Zucker diabetic fatty (ZDF) rat with a point mutation in the leptin receptor is a recognized model of obesity, hyperlipidemia, hyperglycemia and hypertension [28-30]. In the present study, only male rats were used, since female ZDF rats are less prone to the development of metabolic syndrome [31,32]. Male ZDF rats develop an age-dependent obese and hyperglycemic phenotype at 10-12 weeks of age accompanied by a metabolic state of obesity, dyslipidemia, hyperinsulinemia and insulin resistance [33,34] which develops to a hyperglycemic insulin-deficient state [33]. The metabolic features manifested in this animal model are in many ways similar to the pathogenesis of metabolic syndrome in humans [33,35]. Therefore, the ZDF rat is an ideal model for investigation of cardiac gene expression pattern changes related to human metabolic syndrome.

### Experimental setup

**Figure 1:**
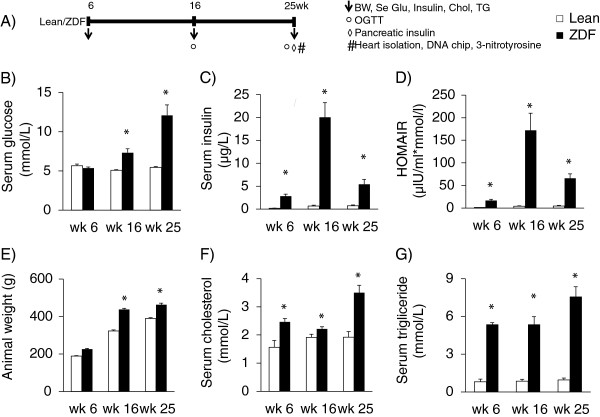
**Experimental protocol (A) Male Zucker Diabetic Fatty (ZDF) rats and their lean controls were followed up from 6 weeks of age until 25 weeks of age.** Body weight (BW), serum glucose (Se Glu), insulin (Insulin), cholesterol (Chol) and triglyceride (TG) levels were determined at week 6, 16 and 25. Oral glucose tolerance test (OGTT) was performed at week 16 and 25. At week 25, hearts and pancreata were isolated. Pancreata were frozen and homogenized to measure pancreatic insulin levels. Hearts were perfused according to Langendorff for 10 minutes using Krebs-Henseleit buffer. Then ventricular tissue was frozen and used for DNA microarray analysis and cardiac free 3-nitrotyrosine level measurements. Serum glucose (**B**, n=6-8) and insulin levels (**C**, n=6-8), HOMA-IR (**D**), animal weight (**E**), serum cholesterol (**F**, n=6-8) and triglyceride (**G**, n=6-8) shown at week 6, 16 and 25 in both lean and ZDF rats. Solid line: Lean; dashed line: ZDF. Values are means±SEM, *p<0.05.

Body weight, serum glucose, insulin, cholesterol and triglyceride levels and homeostasis model assessment-estimated insulin resistance (HOMA-IR) were determined at 6, 16 and 25 weeks of age in order to monitor the basic parameters of glucose and lipid metabolism and insulin resistance in ZDF and lean rats (Figure 1). Oral glucose tolerance test (OGTT) was performed at week 16 and 25 in order to further characterize glucose homeostasis in ZDF and lean rats (Figure 1). At 25 weeks of age, rats were anaesthetized using diethyl ether. Hearts and pancreata were isolated (Figure 1), and then hearts were perfused according to Langendorff as described earlier [36]. After 10 min perfusion ventricular tissue was frozen and stored at -80°C until DNA microarray investigation and gene expression analysis (Figure 1). To validate the well-known nitrosative stress-inducing effect of metabolic syndrome on the heart, frozen ventricular tissue was used for determination of cardiac free 3-nitrotyrosine level (Figure 1).

### Serum glucose level measurements and OGTT

Rats were fasted overnight (12 h) prior to serum glucose level measurements (week 6, 16 and 25) and OGTTs (week16 and 25) to verify the development of hyperglycemia as a diagnostic criterion of metabolic syndrome. Blood samples were collected from the v. saphena. Blood glucose levels were measured using AccuCheck blood glucose monitoring systems (Roche Diagnostics Corporation, USA, Indianapolis). In case of OGTT, after the measurement of baseline glucose concentrations, a standard dose of glucose (1.5 g/kg body weight) was administered per os via gavage and plasma glucose levels were checked 30, 60 and 120 minutes later. Area under the curve values for OGTT was also calculated.

### Measurement of serum and pancreatic insulin levels

Serum and pancreatic insulin levels were measured by an enzyme immunoassay (Mercodia, Ultrasensitive Rat Insulin ELISA) in order to verify the development of hyperinsulinemia and decreased pancreatic insulin content as a consequence of beta cell damage in metabolic syndrome. Insulin ELISA was carried out according to the instructions of the manufacturer from either sera or homogenized pancreatic tissue samples of ZDF and lean control rats. Sera were centrifuged (4500 rpm for 10 min at 4°C) and kept at -20°C until further investigation. Pancreata were removed, trimmed free of adipose tissue and weighed. Pancreata were homogenized in 6 ml cold acidified-ethanol (0.7 M HCl: ethanol (1:3 v/v) with an Ultraturrax homogenizer and were kept at 4°C for 24 h. Then pancreas homogenates were centrifuged (900 g for 15 min at 4°C), and the supernatants were stored at 4°C. The pellet was extracted again with 3 ml acidified ethanol for 24 h at 4°C. The supernatant obtained after centrifugation was pooled with the previous one and kept at -20°C until assayed.

### HOMA-IR index

To estimate insulin resistance in ZDF or lean rats the widely used HOMA-IR index was calculated [37-39] by multiplying fasting plasma insulin (μIU/mL) with fasting plasma glucose (mmol/L), then dividing by the constant 22.5, i.e. HOMA-IR = (fasting plasma insulin concentration×fasting plasma glucose concentration)/22.5.

### Measurement of serum lipid levels

Serum cholesterol and triglyceride levels were measured at week 6, 16 and 25 using a test kit supplied by Diagnosticum Zrt. (Budapest, Hungary) as described previously [40] in order to follow up the development of hyperlipidemia which is a diagnostic criterion of metabolic syndrome.

### Cardiac 3-nitrotyrosine level, an indicator of myocardial nitrosative stress

To verify the well-known increased oxidative/nitrosative stress [41,42] in the heart in metabolic syndrome, cardiac free 3-nitrotyrosine level, an indirect marker of nitrosative stress, was measured by ELISA (Cayman Chemical) from ZDF and lean control heart tissue samples at week 25 as described earlier [40]. Briefly, supernatants of ventricular tissue homogenates were incubated overnight with anti-nitrotyrosine rabbit IgG specific to free 3-nitrotyrosine and nitrotyrosine acetylcholinesterase tracer in precoated (mouse anti-rabbit IgG) microplates followed by development with Ellman's reagent. Free nitrotyrosine content was normalized to protein content of the cardiac homogenate and expressed as nanograms per milligram protein [40].

### RNA preparation

Total RNA was purified from whole heart of Zucker Diabetic Fatty (ZDF) and lean control rats (n=6-8 in each group) using an RNA isolation kit (Macherey-Nagel, Düren, Germany). All the preparation steps were carried out according to the manufacturer’s instructions. RNA samples were stored at –80°C in the presence of 30 U Prime RNAse inhibitor (Fermentas, Lithuania) untill further analysis. The quantity of isolated RNA samples was checked by spectrophotometry (NanoDrop 3.1.0, Rockland, DE, USA).

### DNA microarray analysis

Total RNAs (1 μg) were first reverse transcribed in 10 μl volume using Oligo(dT) Primer and ArrayScript enzyme as described previously [43]. Than the second cDNA strand was synthesized in 50 μl final volume using DNA Polymerase and RNase H. Amino allyl modified aRNA were than synthesized by In Vitro Transcription using aaUTP and T7 Enzyme mix. All these steps were done using AminoAllyl MessageAmpTM II aRNA Amplification Kit (Ambion, USA), according to manufacturer’s instructions. Six μg of amino allyl modified amplified RNA were labeled with either Cy5 or Cy3 dyes in 10 μl volume according to the manufacturer’s instructions (Ambion, USA), than purified using RNA purification columns (Macherey Nagel, Düren, Germany).

Rat microarray of 8-plex format from Agilent Technologies (Palo Alto, CA, USA) was used to determine gene expression changes in the hearts of ZDF rats compared to lean controls. Each matrix contains ~15.000 oligonucleotides corresponding to different genes and control sequences. 300 ng of Cy5 and Cy3 labeled RNA in 19 μl volume, 5 μl 10X Blocking Agent and 1 μl 25X Fragmentation Buffer were mixed together and incubated at 60°C for 30 minutes. 25 μl 2X GEx Hybridization Buffer were added to each sample, to stop the fragmentation reaction. All these steps were done using Gene expression hybridization kit (Agilent Technologies, Palo Alto, CA). 48 μl of these mixes were used for the hybridization, which was done in microarray hybridization chambers (Agilent Technologies, Palo Alto, CA). The chambers were then loaded into a hybridization rotator rack (~5 rpm) and incubated at 65°C for 17 hours. After hybridization the slides were washed in Wash buffer 1 from Agilent Technologies at room temperature for 1 minute than in Wash buffer 2 at 37°C for another 1 minutes before scanning. Each array was scanned at 543 nm (for Cy3 labeling) or at 633 nm (for Cy5 labeling) in Agilent Scanner using the built-in Extended Dynamic Range function with 5 μm resolution. Output image analysis and feature extraction was done using Feature Extraction 9.5.1 software of Agilent Technologies.

### Quantitative real-time PCR (QRT-PCR)

In order to validate gene expression changes obtained by DNA microarray, QRT-PCR was performed on a RotorGene 3000 instrument (Corbett Research, Sydney, Australia) with gene-specific primers and SybrGreen protocol to monitor gene expression as described earlier [44,45]. Briefly, 2 μg of total RNA was reverse transcribed using the High-Capacity cDNA Archive Kit (Applied Biosystems Foster City, CA, USA) according to the manufacturer’s instructions in a final volume of 30 μL. After dilution with 30 μL of water, 1 μL of the diluted reaction mix was used as template in the QRT- PCR with FastStart SYBR Green Master mix (Roche Applied Science, Mannheim, Germany) with the following protocol: 10 min at 95°C followed by 45 cycles of 95°C for 15 sec, 60°C for 25 sec and 72°C for 25 sec. The fluorescence intensity of SybrGreen dye was detected after each amplification step. Melting temperature analysis was done after each reaction to check the quality of the products. Primers were designed using the online Roche Universal Probe Library Assay Design Center. The quality of the primers was verified by MS analysis provided by Bioneer (Daejeon, Korea). Relative expression ratios were calculated as normalized ratios to rat HPRT, GAPDH and Cyclophyllin genes. Non-template control sample was used for each PCR run to check primer-dimer formation. The final relative gene expression ratios were calculated as delta-delta Ct values. Fold change refers to 2^-ΔΔCt^ (in the case of up-regulated genes) and –(1/2^-ΔΔCt^) (in the case of down-regulated genes).

### Gene ontology (GO) analysis

By using DNA microarrays for transcriptional profiling a large number of genes can be analyzed simultaneously [46], however, resulting data do not give direct information about biological interaction of the differentially expressed genes. GO analysis is a suitable method for integration genes with pathways and biological interaction networks to detect coordiated changes in functionally related genes. GO analysis was performed using GO/pathway analysis using the open access software DAVID bioinformatics system and database (Database for Annotation, Visualization and Integrated Discovery, http://david.abcc.ncifcrf.gov). The differentially expressed genes were submitted to DAVID bioinformatics system and database to reveal significantly enriched biological functions/pathways.

### Statistical analysis

For characterization of the ZDF model and lean controls, all values (body weight, serum glucose, insulin, HOMA-IR, cholesterol and triglyceride levels, pancreas weight and insulin content and myocardial 3-nitrotyrosine levels) are presented as mean±SEM. Significance between groups was determined with two sample t-test. P<0.05 was accepted as a statistically significant difference.

In the microarray experiments, dye swap parallel labeling was applied to eliminate dye induced biases. Biological and technical replica experiments were carried out to gain raw data for statistical analysis. Altogether 4 individual parallel gene activity comparisons were done. Statistical analysis was performed to get reliable data. Using two tailed two sample unequal variance Student t-test, the p-value was determined and used to find the significant gene expression changes. Gene expression ratio with p- value < 0.05 and log2 ratio < -0.75 or log2 ratio > 0.75 (~1.7 fold change) are considered as repression or overexpression respectively in gene activity.

## Results

### Characterization of metabolic syndrome

**Figure 2:**
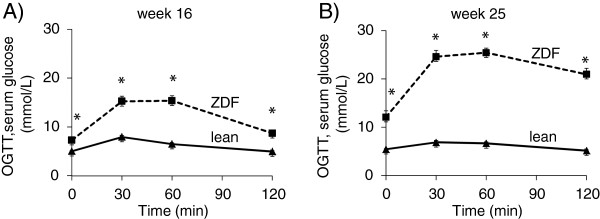
**Glucose levels during OGTT.** Glucose levels during OGTT at week 16 (**A**) and week 25 (**B**) in both lean and ZDF rats. Solid line: Lean; dashed line: ZDF. Values are means±SEM, n=6-8, *p<0.05.

In order to verify the development of metabolic syndrome in male ZDF rats, concentrations of several plasma metabolites and body weight were measured at week 6, 16 and 25 (Figure 1). ZDF rats showed a significant rise in serum fasting glucose level starting from week 16 as compared to lean controls (Figure 1B). Parallel with hyperglycemia, serum insulin levels were significantly increased in ZDF rats compared to lean ones during the 25 weeks showing the presence of hyperinsulinemia in ZDF animals (Figure 1C). However, serum insulin concentration in ZDF rats was significantly lower at week 25 as compared to serum insulin level measured at week 16 indicating beta-cell damage. HOMA IR was significantly higher at week 6, 16 and 25 in ZDF rats when compared to lean controls showing insulin resistance in ZDF animals (Figure 1D). Body weight increased throughout the study and was significantly higher in ZDF animals compared to lean ones showing obesity (Figure 1E). Both serum cholesterol and triglyceride levels were significantly increased in ZDF rats as compared to lean ones throughout the study duration representing hyperlipidemia (Figure 1F and 1G). Oral glucose tolerance test (OGTT) was performed at week 16 and 25 in order to verify the development of impaired glucose tolerance in ZDF rats. Glucose levels during OGTTs were markedly increased in ZDF rats in every time point of blood glucose measurements both at weeks 16 and 25 (Figure 2A- 2B). Area under the curve (AUC) of blood glucose concentration during OGTTs was significantly elevated in ZDF rats at both weeks 16 and 25 (1520±96 vs. 757±13 and 2692±129 vs. 741±21, respectively) representing impaired glucose tolerance.

**Figure 3:**
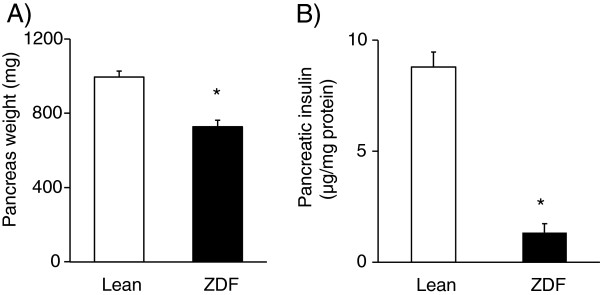
**Pancreas weight and pancreas insulin content.** Pancreas weight (**A**) at week 25 and pancreatic insulin content (**B**) in both lean and ZDF rats. Values are means±SEM, n=6-8, *p<0.05.

Pancreas weight and pancreatic insulin content were measured at the end of the experiment in order to investigate the severity of diabetes mellitus in ZDF rats. Pancreas weight and pancreatic insulin concentration were significantly decreased in ZDF rats at week 25 showing impaired pancreatic function (Figure 3A and 3B).

**Figure 4:**
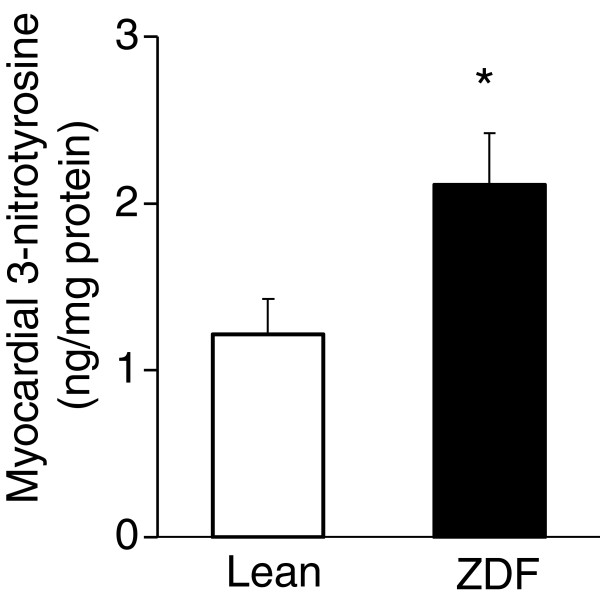
**Myocardial 3-nitrotyrosine level.** Myocardial 3-nitrotyrosine level at week 25 in both lean and ZDF rats. Values are means±SEM, *p<0.05.

In order to verify the increased oxidative/nitrosative stress in ZDF animals, myocardial 3-nitrotyrosine levels were determined in both groups at week 25. A marker molecule of peroxynitrite, 3-nitrotyrosine level was significantly elevated in the heart of ZDF animals showing increased cardiac oxidative/nitrosative stress (Figure 4).

### Gene expression profiles measured using cDNA microarrays and by qRT-PCR

Among the 14921 genes surveyed, 10244 genes were expressed on the cDNA microarray, and 85 genes whose expression was > ~1.7-fold up- or down-regulated (log2 ratio <-0.75 or log2 ratio >0.75) in hearts of ZDF rats relative to levels of lean control rats showed significant change in expression. According to our results, 49 genes showed down-regulation (Table 1) and 36 genes showed up-regulation in hearts of ZDF rats (Table 2). The expression change of selected 23 genes was validated by qRT-PCR (Table 3 and 4), 18 of these 23 genes have been confirmed by qRT-PCR (Table 3). Most of these genes have not been shown to be involved in the development of cardiovascular complications of metabolic syndrome yet.

**Table 1:** Down-regulated genes on DNA microarray

**Gene function**	**Description [Gene symbol]**	**Acc. No.:**	**AVE log**_**2**_	**SD**	**P value**	**Fold change**
**Metabolism**	Argininosuccinate synthetase [Ass]	NM_013157	−1.33	0.06	0.001	−2.51
	Glycine C-acetyltransferase (2-amino-3-ketobutyrate-coenzyme A ligase) [Gcat]	NM_001024277	−1.15	0.34	0.006	−2.22
	3-hydroxybutyrate dehydrogenase, type 1 [Bdh1]	NM_053995	−1.14	0.44	0.014	−2.21
	Thymidylate synthase [Tyms]	NM_019179	−0.92	0.08	0.041	−1.89
	Dicarbonyl L-xylulose reductase [Dcxr]	NM_134387	−0.76	0.30	0.015	−1.70
**Stress response**	Heat shock 70kd protein 1A [Hspa1a]	NM_031971	−1.84	0.48	0.005	−3.59
	Similar to 60 kDa heat shock protein, mitochondrial precursor (Hsp60) 60 kda heat shock protein [LOC294396]	XR_085659	−1.64	0.24	0.057	−3.11
	Interleukin 18 [il18]	NM_019165	−1.38	0.02	0.007	−2.60
**Signal transduction, regulation of transcription**	Hairy/anhancer-of-split-related with YRPW motif2 [Hey2]	NM_130417	−1.40	0.15	0.049	−2.64
	Aryl hydrocarbon receptor nuclear translocator-like [Adra1d]	NM_024362	−1.33	0.07	0.001	−2.52
	Activating transcription factor 3 [Atf3]	NM_012912	−1.03	0.26	0.004	−2.05
	RAB7, member RAS oncogene family [Rab7]	NM_023950	−0.98	0.48	0.026	−1.97
	B-cell leukemia/lymphoma 2 related protein A1 [Bcl2a1]	NM_133416	−0.89	0.04	0.019	−1.85
	Sushi, nidogen and EGF-like domains 1 [Sned1]	XM_237415	−0.87	0.51	0.041	−1.83
	Spermatid perinuclear RNA binding protein [Strbp]	NM_053416	−0.87	0.25	0.027	−1.82
	TRAF3 interacting protein 3 [Traf3ip3]	NM_001014132	−0.85	0.25	0.028	−1.81
	Putative bhlh transcription factor (Fragment) [Ascl3]	ENSRNOT00000018309	−0.79	0.15	0.013	−1.73
**Membrane proteins, receptors**	Atpase, (Na+)/K+ transporting, beta 4 polypeptide [Atp1b4]	NM_053381	−1.38	0.13	0.003	−2.60
	Nerve growth factor receptor (TNFR superfamily, member 16) [Ngfr]	NM_012610	−0.91	0.05	0.027	−1.88
	Cholinergic receptor, nicotinic, gamma polypeptide [Chrng]	NM_019145	−0.99	0.39	0.048	−1.98
	G protein-coupled receptor 37 [Gpr37]	NM_057201	−0.87	0.06	0.030	−1.82
	Adrenergic receptor, alpha 1d [Adra1d]	NM_024483	−0.79	0.43	0.036	−1.73
	Neurotensin receptor 2 [Ntsr2]	NM_022695	−0.77	0.32	0.018	−1.70
**Structural protein, cell adhesion**	Myosin ixa [myo9a]	NM_134335	−1.26	0.01	0.002	−2.40
	ST8 alpha-N-acetyl-neuraminide alpha-2,8-sialyltransferase 4 [St8sia4]	NM_053914	−1.00	0.07	0.032	−2.01
	Similar to collagen, type XXIV, alpha 1 [RGD1565539_predicted]	XM_575056	−1.16	0.03	0.011	−2.24
**Others**	ADAM metallopeptidase with thrombospondin type 1 motif, 1 [Adamts1]	NM_024400	−2.84	0.90	0.1230	−7.16
	Similar to tatd dnase domain containing 1 [RGD1566244_predicted]	XR_007720	−1.86	0.16	0.038	−3.63
	Aryl hydrocarbon receptor nuclear translocator-like protein [Arntl]	NM_024362	−1.33	0.67	0.001	−2.52
	TAF13 RNA polymerase II, TATA box binding protein (TBP)-associated factor [Taf13_predicted]	ENSRNOT00000027530	−1.22	0.11	0.039	−2.34
	G protein-coupled receptor 107 [Gpr107_predicted]	ENSRNOT00000037742	−1.18	0.02	0.007	−2.26
	CDK5 regulatory subunit associated protein 1-like 1 [Cdkal1_predicted]	XM_341524	−0.92	0.06	0.041	−1.89
	Similar to chondroitin beta1,4 N-acetylgalactosaminyltransferase 2 [RGD1563660_predicted]	ENSRNOT00000019778	−0.91	0.03	0.017	−1.87
	S100 calcium binding protein A3 [S100a3]	NM_053681	−0.90	0.06	0.028	−1.87
	Similar to TPR repeat-containing protein KIAA1043 [LOC304558]	XM_222260	−0.89	0.08	0.003	−1.85
	Similar to Ten-m4 [Odz4_predicted]	ENSRNOT00000015181	−0.88	0.34	0.047	−1.84
	Kallikrein 14 [Klk14_predicted]	ENSRNOT00000029197	−0.88	0.09	0.047	−1.84
	Mucin 19 [Muc19_predicted]	XM_235593	−0.84	0.33	0.048	1.79
	Copine family member IX [Cpne9]	NM_001024982	−0.84	0.11	0.006	−1.79
	Suppressor of Ty 16 homolog (S. Cerevisiae) [Supt16h_predicted]	XM_223981	−0.81	1.57	0.021	−1.75
	Heterogeneous nuclear ribonucleoprotein L-like [Hnrpll_predicted]	XM_233805	−0.81	0.01	0.008	−1.75
	Cdna clone UI-R-BJ0p-afn-b-03-0-UI 3' [Sln]	CK841541	−0.81	0.30	0.013	−1.75
	Discs, large homolog 4 (Drosophila) [Dlgh4]	NM_019621	−0.80	0.23	0.026	−1.75
	Similar to chondroitin beta1,4 N-acetylgalactosaminyltransferase [RGD1307618_predicted]	XM_224757	−0.80	0.25	0.031	−1.74
	Sterile alpha motif domain containing 4 [Samd4_predicted]	ENSRNOT00000060847	−0.80	0.02	0.012	−1.74
	Similar to RIKEN cdna 1190005B03 [Cdkal1_predicted]	ENSRNOT00000024854	−0.79	0.06	0.035	−1.73
	WDNM1 homolog [LOC360228]	NM_001003706	−0.78	0.24	0.007	−1.72
	CD300 antigen like family member E [Cd300le_predicted]	XR_009489	−0.78	0.362	0.023	−1.71
	Neuronatin [nnat]	NM_053601	−0.77	0.35	0.022	−1.71
	Connective tissue growth factor [Ctgf]	NM_022266	−0.77	0.47	0.049	−1.69
	Chemokine (C-X-C motif) ligand 11 [Cxcl11]	NM_182952	−0.75	0.17	0.003	−1.68

**Table 2:** Up-regulated genes on DNA microarray

**Gene function**	**Description [**Gene symbol**]**	**Acc. No.:**	**AVE log**_**2**_	**SD**	**P value**	**Fold change**
**Metabolism**	Acyl-coa thioesterase 7 [Acot7]	NM_013214	0.75	0.34	0.021	1.69
	Angiopoietin-like 4 [Angptl4]	NM_199115	0.83	0.25	0.007	1.78
	Mannosyl (alpha-1,3-)-glycoprotein beta-1,4-N-acetylglucosaminyltransferase, isozyme C [Mgat4c_predicted]	ENSRNOT00000005523	0.87	0.29	0.037	1.82
	Carbonyl reductase 1 [Cbr1]	NM_019170	0.99	0.40	0.016	1.99
	3-hydroxy-3-methylglutaryl-Coenzyme A synthase 2 [Hmgcs2]	NM_173094	1.05	0.25	0.004	2.07
	Transglutaminase 1 [tgm1]	NM_031659	1.35	0.13	0.044	2.55
	Cytosolic acyl-coa thioesterase 1 [Cte1]	NM_031315	1.88	0.41	0.003	3.69
**Stress response**	Cold inducible RNA binding protein [Cirbp]	NM_031147	0.77	0.21	0.005	1.71
	Glutathione S-transferase Yc2 subunit [Yc2]	NM_001009920	0.86	0.16	0.002	1.82
**Signal transduction, regulation of transcription**	Calcium/calmodulin-dependent protein kinase II gamma [Camk2g]	NM_133605	0.75	0.21	0.006	1.68
	Phospholipase A2, group VII (platelet-activating factor acetylhydrolase, plasma) [Pla2g7]	NM_001009353	1.45	0.84	0.041	2.74
	Brain expressed X-linked 1 [Bex1]	NM_001037365	0.92	0.57	0.048	1.90
	Fibroblast growth factor receptor substrate 3 [Frs3]	NM_001017382	1.05	0.04	0.018	2.07
**Membrane proteins, receptors**	Huntingtin-associated protein 1 [Hap1]	NM_024133	0.77	0.29	0.043	1.71
	Membrane protein, palmitoylated 3 (MAGUK p55 subfamily member 3) [RGD1560049_predicted]	ENSRNOT00000055194	1.10	0.45	0.016	2.15
	Atpase, H+/K+ transporting, nongastric, alpha polypeptide [Atp12a]	NM_133517	1.39	0.19	0.006	2.61
**Structural protein, cell adhesion**	Spectrin beta 3 [Spnb3]	NM_019167	0.95	0.05	0.026	1.93
	Aggrecan 1 [agc1]	NM_022190	1.06	0.09	0.037	2.08
**Others**	Similar to mucin 7, salivary [RGD1311530_predicted]	ENSRNOT00000014519	0.75	0.22	0.007	1.69
	Leukocyte tyrosine kinase [Ltk_predicted]	ENSRNOT00000050055	0.77	0.04	0.021	1.70
	Similar to RIKEN cdna 9130022B02 [Pck2_predicted]	ENSRNOT00000025260	0.78	0.28	0.042	1.72
	Similar to hypothetical protein [Arid2_predicted]	ENSRNOT00000006970	0.79	0.20	0.004	1.73
	Iroquois related homeobox 3 (Drosophila) [Irx3_predicted]	ENSRNOT00000043254	0.809	0.41	0.031	1.74
	Hdac5 protein (Fragment) [Hdac5]	ENSRNOT00000028381	0.86	0.70	0.022	1.81
	Ribonuclease, rnase A family, 1 (pancreatic) [Rnase1]	NM_001029904	0.90	0.22	0.004	1.86
	Ring finger protein 24 [Rnf24_predicted]	ENSRNOT00000028869	0.92	0.22	0.037	1.89
	Amyloid beta (A4) precursor protein-binding, family A, member 1 [Apba1]	NM_031779	0.96	0.36	0.044	1.94
	CWF19-like 1, cell cycle control (S. Pombe) [Cwf19l1_predicted]	ENSRNOT00000017202	0.97	0.09	0.044	1.95
	Similar to high density lipoprotein-binding protein [RGD1564237_predicted]	ENSRNOT00000009811	0.98	0.19	0.002	1.97
	P21 (CDKN1A)-activated kinase 6 [Pak6_predicted]	ENSRNOT00000010471	0.10	0.08	0.036	2.00
	Similar to nuclear body associated kinase 1a [Hipk2_predicted]	XM_342662	1.06	0.66	0.049	2.09
	Cationic trypsinogen [LOC286911]	NM_173127	1.08	0.07	0.031	2.11
	Chac, cation transport regulator-like 1 (E. Coli) [RGD1560049_predicted]	XM_342497	1.10	0.08	0.033	2.15
	NTAK alpha2 [Nrg2]	D89996	1.13	0.10	0.042	2.18
	Ubiquitin carboxy-terminal hydrolase L1 [Uchl1]	NM_017237	1.24	0.26	0.014	2.37
	Claudin 19 [cldn19]	NM_001008514	1.43	0.12	0.037	2.70

**Table 3:** QRT-PCR

**Description [Gene symbol]**	**Acc. No.:**	**DNA MICROARRAY**		**QRT-PCR**			**Confirmed**
		**Fold change**	**p value**	**Ratio (SD)**	**Fold change**	**Regulation**	
ADAM metallopeptidase with thrombospondin type 1 motif, 1 [Adamts1]	NM_024400	**−7.16**	0.1295	0.58 (0.09)	**−1.72**	down	***yes***
heat shock 70kD protein 1A [Hspa1a]	NM_031971	**−3.59**	0.0045	0.34 (0.06)	**−2.94**	down	***yes***
similar to 60 kDa heat shock protein mitochondrial precursor [Hsp60]	XR_085659	**−3.11**	0.0565	0.64 (0.10)	**−1.56**	down	***yes***
interleukin 18 [Il18]	NM_019165	**−2.60**	0.0066	0.91 (0.15)	**−1.10**	no change	no
ATPase, (Na+)/K+ transporting, beta 4 polypeptide [Atp1b4]	NM_053381	**−2.60**	0.0031	0.37 (0.06)	**−2.70**	down	***yes***
argininosuccinate synthetase [Ass]	NM_013157	**−2.51**	0.0007	0.29 (0.05)	**−3.42**	down	***yes***
myosin IXA [Myo9a]	NM_134335	**−2.40**	0.0018	0.90 (0.15)	**−1.11**	no change	no
glycine C-acetyltransferase (2-amino-3-ketobutyrate-coenzyme A ligase) [Gcat]	NM_001024277	**−2.22**	0.0063	0.33 (0.03)	**−3.07**	down	***yes***
activating transcription factor 3 [Atf3]	NM_012912	**−2.05**	0.0044	0.35 (0.06)	**−2.85**	down	***yes***
similar to chondroitin sulfate GalNAcT-2 [RGD1563660_predicted]	ENSRNOT00000019778	**−1.87**	0.0170	0.92 (0.15)	**−1.09**	no change	no
S100 calcium binding protein A3 [S100a3]	NM_053681	**−1.87**	0.0281	1.74 (0.28)	**1.74**	up	no
sushi, nidogen and EGF-like domains 1 [Sned1]	NM_001167842	**−1.83**	0.0407	0.42 (0.07)	**−2.36**	down	***yes***
G protein-coupled receptor 37 [Gpr37]	NM_057201	**−1.82**	0.0297	1.08 (0.18)	**1.08**	no change	no
angiopoietin-like 4 [Angptl4]	NM_199115	**1.78**	0.0073	2.90 (0.47)	**2.90**	up	***yes***
glutathione S-transferase Yc2 subunit [Yc2]	NM_001009920	**1.82**	0.0017	2.21 (0.36)	**2.21**	up	***yes***
ribonuclease, RNase A family, 1 (pancreatic) [Rnase1]	NM_001029904	**1.86**	0.0039	2.68 (0.44)	**2.68**	up	***yes***
similar to high density lipoprotein-binding protein [RGD1564237_predicted]	ENSRNOT00000009811	**1.97**	0.0020	2.73 (0.44)	**2.73**	up	***yes***
3-hydroxy-3-methylglutaryl-Coenzyme A synthase 2[Hmgcs2]	NM_173094	**2.07**	0.0036	2.42 (0.39)	**2.42**	up	***yes***
similar to tetracycline transporter-like protein [RGD1311900_predicted]	ENSRNOT00000017386	**2.24**	0.1671	1.29 (0.21)	**1.29**	no change	no
ubiquitin carboxy-terminal hydrolase L1 [Uchl1]	NM_017237	**2.37**	0.0144	2.50 (0.41)	**2.50**	up	***yes***
ATPase, H+/K+ transporting, nongastric, alpha polypeptide [Atp12a]	NM_133517	**2.61**	0.0061	3.06 (0.50)	**3.06**	up	***yes***
phospholipase A2, group VII (platelet-activating factor acetylhydrolase, plasma) [Pla2g7]	NM_001009353	**2.74**	0.0411	4.25 (0.69)	**4.25**	up	***yes***
cytosolic acyl-CoA thioesterase 1 [Cte1]	NM_031315	**3.69**	0.0028	3.16 (0.51)	**3.16**	up	***yes***

**Table 4:** Primers to QRT-PCR

**Gene symbol**	**Gene description**	**Acc. No.:**	**Forward**	**Reverse**
Adamts1	ADAM metallopeptidase with thrombospondin type 1 motif, 1	NM_024400	aaaggcattggctacttctttg	ggactacagggagtgccatc
Hspa1a	heat shock 70kD protein 1A (Hspa1a)	NM_031971	tggcccattaaataagaaccaa	cgaaggcgtagagattccag
Hsp60	similar to 60 kDa heat shock protein, mitochondrial precursor (Hsp60)	XR_085659	gctacaatttctgcaaacagagac	cattaggggttttcccatcc
Il18	interleukin 18 (Il18)	NM_019165	gcctgatatcgaccgaaca	ccttccatccttcacagatagg
Atp1b4	ATPase, (Na+)/K+ transporting, beta 4 polypeptide (Atp1b4)	NM_053381	acttggcagcgttatgtcatt	catttcctcttgaagactgtcattat
Ass	argininosuccinate synthetase (Ass)	NM_013157	ccaccggcttcatcaatatc	tgctctgaaggcgatggta
Myo9a	myosin IXA (Myo9a)	NM_134335	cactctgagctagggcctgt	actgaagaaaatcgttgtgacg
Gcat	glycine C-acetyltransferase (2-amino-3-ketobutyrate-coenzyme A ligase) (Gcat)	NM_001024277	gctggcctcatttctactcg	gcgggctatcttggcttc
Atf3	activating transcription factor 3	NM_012912	tgtcagtcaccaagtctgaggt	cacttggcagcagcaattt
RGD1563660_pred	similar to chondroitin sulfate GalNAcT-2 (pred)	NM_001106616	tcgtctatgccaaccagga	tctccaaaaaccagagtccttt
S100a3	S100 calcium binding protein A3	NM_053681	agcagcagcagcagttga	ggtacacacgatggcagcta
Sned1	sushi, nidogen and EGF-like domains 1 (Sned1)	NM_001167842	cctggtaccgtgtgaccttc	caccgtttggaatgtgttga
Gpr37	G protein-coupled receptor 37	NM_057201	ccaagaagtggcttttggaa	agtgacacccagagaagctacc
Angptl4	angiopoietin-like 4	NM_199115	tctccaccatttttggtcaac	gttcaggcgtctctgaatcac
Yc2	glutathione S-transferase Yc2 subunit	NM_001009920	tctgaaaactcgggatgacc	accagcttcatcccgtca
Rnase1	ribonuclease, RNase A family, 1 (pancreatic)	NM_001029904	actgactgccgcctgaag	ttctggctgtcagtggttgt
RGD1311900_pred RGD	similar to tetracycline transporter-like protein	ENSRNOT00000017386	gcactcactgcctatgttgg	cctggagaaccatagctgga
RGD1564237_pred RGD	similar to high density lipoprotein-binding protein	ENSRNOT00000009811	ggaggagaccaacatgatcc	agcacttggcagaagtagcac
Hmgcs2	3-hydroxy-3-methylglutaryl-Coenzyme A synthase 2	NM_173094	cctggcctcacttctctcc	ggagaaggctccaatcctg
Uchl1	ubiquitin carboxy-terminal hydrolase L1	NM_017237	attcaggcagcccatgact	gaaattcactttgtcgtctaccc
Atp12a	ATPase, H+/K+ transporting, nongastric, alpha polypeptide	NM_133517	gcatcattgtggctaacgtg	ccgtcagtgacagggtaaca
Pla2g7	phospholipase A2, group VII (platelet-activating factor acetylhydrolase	NM_001009353	actggcaagacccttcttttt	gacatcaccgattggagctt
Cte1	cytosolic acyl-CoA thioesterase 1	NM_031315	gtgcacgagcgtcacttc	gaaagggcccaggttctg

### Gene ontology analysis

In order to further determine the biological significance and functional classification of differentially expressed genes due to metabolic syndrome, GO analysis was performed (Table 5). GO is a bioinformatics initiative with the aim of standardizing the representation of genes and gene products providing a controlled and regularly updated vocabulary of terms for gene product characteristics and annotation data. GO analysis is suitable for identifying significantly enriched GO terms related to multiple genes and for discovering enriched functionally related gene groups. A single gene can belong to different categories. Out of the 85 genes significantly altered by metabolic syndrome in our present study, 68 genes with known function were submitted to GO analysis. The rest of the 85 genes were either unknown expressed sequence tags or unrecognized by the GO analysis database. The 68 analyzed genes were classified into three main categories such as (i) cellular metabolic process, (ii) developmental process and (iii) localization including transport (Table 5).

**Table 5:** Gene ontology analysis

**Category**	**GO ID**	**Level**	**Term**	**Count**	**%**	**p value**	**Genes (gene symbols)**
GOTERM_BP_ALL	GO:0009987	02	cellular process	47	55,3	0,035	ACAN, ACOT1, ACOT7, ANGPTL4, APBA1, ARID2, ARNTL, ASS1, ATF3, ATP1B4, ATP12A,BCL2A1D, BEX1, CAMK2G, CBR1, CHRNG, COL24A1, CSGALNACT1, CTGF, DCXR, DLG4, DUSP3, EXO1, GSTA5, HAP1, HDAC5, HEY2, HIPK2, HMGCS2, HSPA1A, HSPA1B, HNRPLL, IRX3, LOC360228, LOC501189, NGFR, NNAT, NTSR2, PCK2, SNED1, SPTBN2, ST8SIA4, STRBP, TAF13, TGM1, TYMS, UCHL1
GOTERM_BP_ALL	GO:0009266	04	response to temperature stimulus	5	5,9	0,013	CIRBP, HSPA1A, HSPA1B, IL18, NGFR
GOTERM_BP_ALL	GO:0042180	04	cellular ketone metabolic process	9	10,6	0,004	ACOT1, ACOT7, ARID2, ASS1, ATF3, CBR1, CSGALNACT1, HNRPLL, PCK2
GOTERM_BP_ALL	GO:0006082	04	organic acid metabolic process	8	9,4	0,014	ACOT1, ACOT7, ARID2, ASS1, ATF3, CSGALNACT1, HNRPLL, PCK2
GOTERM_BP_ALL	GO:0019752	05	carboxylic acid metabolic process	8	9,4	0,014	ACOT1, ACOT7, ARID2, ASS1, ATF3, CSGALNACT1, HNRPLL, PCK2
GOTERM_BP_ALL	GO:0043436	05	oxoacid metabolic process	8	9,4	0,014	ACOT1, ACOT7, ARID2, ASS1, ATF3, CSGALNACT1, HNRPLL, PCK2
GOTERM_BP_ALL	GO:0034637	05	cellular carbohydrate biosynthetic process	3	3,5	0,039	ATF3, CSGALNACT1, PCK2
GOTERM_BP_ALL	GO:0051346	06	negative regulation of hydrolase activity	4	4,7	0,023	ANGPTL4, BCL2A1D, HSPA1A, HSPA1B,
GOTERM_BP_ALL	GO:0008284	06	positive regulation of cell proliferation	6	7,1	0,039	ATF3, BEX1, HEY2, HIPK2, IL18, NGFR
GOTERM_BP_ALL	GO:0048489	07	synaptic vesicle transport	3	3,5	0,017	APBA1, DLG4, SPTBN2
GOTERM_BP_ALL	GO:0032502	02	developmental process	25	29,4	0,001	ACAN, ANGPTL4, APBA1, ASS1, ATF3, BCL2A1D, BEX1, CBR1, CTGF, DLG4, EXO1, HAP1, HEY2, HIPK2, IL18, IRX3, KLK14, LOC360228, NGFR, NNAT, NRG2, ODZ4, STRBP, TGM1, UCHL1
GOTERM_BP_ALL	GO:0048869	03	cellular developmental process	15	17,6	0,012	ACAN, ANGPTL4, ATF3, BCL2A1D, BEX1, CTGF, DLG4, HEY2, IRX3, LOC360228, NGFR, NNAT, STRBP, TGM1, UCHL1
GOTERM_BP_ALL	GO:0007275	03	multicellular organismal development	23	27,1	0,001	ACAN, ANGPTL4, APBA1, ASS1, BCL2A1D, BEX1, CBR1, CTGF, DLG4, EXO1,HEY2, HAP1, HIPK2, IL18, IRX3, KLK14, NGFR, NNAT, NRG2, ODZ4, STRBP, TGM1, UCHL1
GOTERM_BP_ALL	GO:0048856	03	anatomical structure development	19	22,3	0,013	ACAN, ANGPTL4, ASS1, BCL2A1D, BEX1, CBR1, CTGF, DLG4, EXO1, HAP1, HEY2, IL18, IRX3, KLK14, NGFR, NNAT, ODZ4, TGM1, UCHL1
GOTERM_BP_ALL	GO:0048731	04	system development	18	21,1	0,016	ACAN, ANGPTL4, ASS1, BCL2A1D, BEX1, CBR1, CTGF, DLG4, EXO1, HAP1, HEY2, IL18, IRX3, KLK14, NGFR, NNAT, TGM1, UCHL1
GOTERM_BP_ALL	GO:0030154	04	cell differentiation	15	17,6	0,009	ACAN, ANGPTL4 ATF3, BCL2A1D, BEX1, CTGF, DLG4, HEY2, IRX3, LOC360228, NGFR, NNAT, STRBP, TGM1, UCHL1
GOTERM_MF_ALL	GO:0003824	02	catalytic activity	30	35,3	0,037	ACOT1, ACOT7, ARID2, ASS1, ATP1B4, ATP12A ,BDH1, CAMK2G, CBR1, CSGALNACT1, DCXR, DUSP3, EXO1,GCAT, GSTA5, HDAC5, HIPK2, HMGCS2, KLK14, LOC286911, LOC501189, MYO9A, PCK2, PLA2G7, RAB7A, RNASE1, ST8SIA4, TYMS, TGM1, UCHL1
GOTERM_MF_ALL	GO:0016788	04	hydrolase activity, acting on ester bonds	7	8,2	0,041	ACOT1, ACOT7, DUSP3, EXO1, PLA2G7, RNASE1, UCHL1
GOTERM_MF_ALL	GO:0016790	05	thiolester hydrolase activity	3	3,5	0,047	ACOT1, ACOT7, UCHL1
GOTERM_CC_ALL	GO:0031974	02	membrane-enclosed lumen	13	14,5	0,032	ARNTL, ASCL3, ASCL3_PREDICTED, ATF3, BDH1, CIRBP, DUSP3, HIPK2, HMGCS2, NGFR, SPTBN2, SUPT16H, TAF13
GOTERM_CC_ALL	GO:0044421	03	extracellular region part	8	9,6	0,044	ACAN, ANGPTL4, COL24A1, CTGF, LOC286911, LOC360228, IL18, KLK14
GOTERM_CC_ALL	GO:0031090	04	organelle membrane	11	12,0	0,032	ASS1, CAMK2G, CSGALNACT1, BDH1, DLG4, GCAT, GSTA5, HIPK2, HMGCS2, LOC501189, ST8SIA4
GOTERM_CC_ALL	GO:0043233	04	organelle lumen	13	15,3	0,027	ARNTL, ASCL3, ASCL3_PREDICTED, ATF3, BDH1, CIRBP, DUSP3, HIPK2, HMGCS2, NGFR, SPTBN2, SUPT16H, TAF13
GOTERM_CC_ALL	GO:0070013	06	intracellular organelle lumen	13	15,3	0,021	ARNTL, ASCL3, ASCL3_PREDICTED, ATF3, BDH1, CIRBP, DUSP3, HIPK2, HMGCS2, NGFR, SPTBN2, SUPT16H, TAF13
GOTERM_CC_ALL	GO:0005654	06	nucleoplasm	9	10,6	0,045	ARNTL, ASCL3, ASCL3_PREDICTED, CIRBP, DUSP3, HIPK2, NGFR, SUPT16H, TAF13
GOTERM_CC_ALL	GO:0031981	07	nuclear lumen	12	14,1	0,029	ARNTL, ASCL3, ASCL3_PREDICTED, ATF3, CIRBP, DUSP3, HIPK2, NGFR, SPTBN2, SUPT16H, TAF13
GOTERM_CC_ALL	GO:0030139	08	endocytic vesicle	3	3,5	0,034	CAMK2G, DLG4, RAB7A

Significantly enriched gene ontology (GO) terms in the population of genes with altered expression due to metabolic syndrome. GO analysis determines the biological significance of differentially expressed genes that can be used to determine the functional classification of the genes, the expression of which have been significantly up- or down-regulated. Major functional categories of GO terms were separated by horizontal lines, subcategories are represented by level on gene tree.

## Discussion

In the present study, our aim was to investigate whether cardiac gene expression is influenced by metabolic syndrome. Here we show several characteristics of metabolic syndrome in 25 weeks old male ZDF rats including obesity, fasting hyperglycemia, hyperlipidemia, hyperinsulinemia, insulin resistance, and impaired glucose tolerance as well as increased cardiac nitrosative stress. In the present study, we demonstrate for the first time in the literature that metabolic syndrome influences cardiac gene expression pattern by altering transcript levels of several genes. We identified 85 genes which were differentially expressed dominantly in the myocardium [47,48] of ZDF rats compared to normal controls. Many of these differentially expressed genes are known to be involved in multiple cell functions, including metabolism, stress response, signal transduction, regulation of transcription, cytoskeletal structure, cell adhesion, membrane proteins, receptors and others. The majority of these genes have not been related to metabolic syndrome yet, and therefore, characterization of the functional effects of these genes on the heart in metabolic syndrome is suggested in future mechanistic studies.

Our present findings showing that 25 week old male ZDF rats develop insulin resistance with hyperinsulinemia, hyperglycemia and impaired HOMA-index are in accordance with previous studies [32-34,49,50]. Both metabolic syndrome and type 2 diabetes mellitus are associated with insulin resistance, hyperinsulinemia and hyperglycemia. Insulin resistance has been reported to be influenced by certain genetic factors and nutrients in patients suffering from metabolic syndrome [51]. It has been proposed that myocardial SERCA2a overexpression stimulated by hyperinsulinemia plays an important role in the cardiac adaptation in ZDF animals [52]. Others have shown that GLUT4 content decreases along with the development of insulin resistance in the myocardium and other insulin sensitive tissues which might play a key role in the impaired glycemic homeostasis in metabolic syndrome [53]. Interestingly, hyperglycemia has been reported to activate p53 and p53-regulated genes involving the local renin-angiotensin system which leads to increased apoptosis of cardiomyocytes [54]. Moreover, postprandial hyperglycemia has been shown to play an important role on the onset and development of heart failure in humans [55]. Chronic hyperglycemia has been reported to enhance the vasoconstrictor response by Rho-kinase [56]. Hyperglycemia itself has been shown to increase rat aortic smooth muscle cell growth and gene expression in diabetes mellitus [57]. Some drugs e.g. statins [57] and nitrates [58] have been reported to abolish hyperglycemia induced vasoconstriction. These aforementioned studies, in agreement with our present study, suggest that metabolic serum parameters may influence cardiac gene expression pattern and may lead to functional consequences. Although we have not measured blood pressure in our study, ZDF rats are well known to develop elevated blood pressure at ages similar to that of used in the present study [59-61]

In our present study, several genes related to metabolism were found to be affected in the hearts of ZDF rats as compared to controls. A group of these altered genes is involved in ketone body metabolism (down-regulation of *3-hydroxybutyrate dehydrogenase, type 1;* up-regulation of *2-amino-3-ketobutyrate-coenzyme A ligase and 3-hydroxy-3-methylglutaryl-coenzyme A synthase 2).* Decreased rate of ketone body oxidation and decreased activity of 3-hydroxybutyrate dehydrogenase activity in streptozotocin-induced diabetic rat hearts have been shown previously [62]. In our present study, metabolic syndrome also influenced expression of genes related to metabolism of carbohydrates (down-regulation of *dicarbonyl L-xylulose reductase* and up-regulation of *mannosyl (alpha-1,3-)-glycoprotein beta-1,4-N-acetylglucosaminyltransferase, isozyme C, pred)* as well as lipids (up-regulation of *acyl-CoA thioesterase 1* and *cytosolic acyl-CoA thioesterase 1*). Similarly to our present findings, gene expression of cytosolic acyl-CoA thioesterase 1 has been reported to be up-regulated by high fat diet [63] or STZ-induced diabetes [63] in the rat myocardium. A third group of differentially expressed metabolic genes in our present study (down-regulation of *argininosuccinate synthetase and* up-regulation of *angiopoietin-like 4)* in ZDF hearts is potentially regulated by oxidative and nitrosative stress which is increased in metabolic diseases e.g. hyperlipidemia [13,40,64], hypertension [65], insulin resistance [66], diabetes mellitus [67] and in the heart of ZDF rats as shown in previous [42] as well as in the present study. High TNF-alpha concentrations [68] and insulin resistance [69,70] in endothelial cells have been reported to reduce the expression of the arginine recycling enzyme, *argininosuccinate synthetase*. Overexpression of hepatic angiopoietin-like 4 gene has been shown in diabetic mice [71] and up-regulation of this gene has reported to be induced by fatty acids via PPAR-gamma in muscle tissue [72]. Additionally, insulin has been shown to down regulate angiopoietin-like 4 in adipocytes [73] and this down-regulation could be attenuated in insulin resistance [73].

Members of another functional gene cluster that is related to stress response showed altered expression in ZDF hearts as compared to controls in the present study (down-regulation of *heat shock 70 kDa protein 1A; similar to 60 kDa heat shock protein, mitochondrial precursor*; *interleukin 18* and up-regulation of *cold inducible RNA binding protein; glutathione S-transferase Yc2 subunit*). We have previously shown that hyperlipidemia inhibits cardiac heat shock response [36]. Moreover, heat shock proteins, especially Hsp60, were found to have protective effect against cardiac oxidative and nitrosative stress [74]. According to the attenuated expression of heat shock protein 60 and 70 in our present study, metabolic syndrome with well-known increased cardiovascular oxidative and nitrosative stress [41,42] due to hyperlipidemia [64], hypertension [65] and hyperglycemia [66,67] might interfere with cardiac heat shock response. Glutathion S-transferase catalyzes the conjugation of reduced glutathione on a wide variety of substrates [75] including reactive oxygen and nitrogen species [76]. Interestingly, we have found here the overexpression of glutathione S-transferase in metabolic syndrome similarly to the up-regulation of this gene in cholesterol diet-induced hyperlipidemia in our previous study [77]. Additionally, the absence of cardiomyopathy in diabetes has been reported to be accompanied by increased glutathione S-transferase activity in rat hearts [78]. These results suggest that up-regulation of glutathione S-transferase may be an adaptive response in metabolic syndrome to antagonize elevated oxidative/nitrosative stress in the myocardium. Elevated circulating interleukin 18 levels have been reported to be associated with metabolic syndrome independent of obesity and insulin resistance [79], however, in our present study; the myocardial gene expression of interleukin 18 was down-regulated.

In the present study, we have also shown altered expression of several genes related to signal transduction and regulation of transcription in the hearts of ZDF rats as compared to controls (e.g. down-regulation of *activating transcription factor 3*; *sushi, nidogen and EGF-like domains 1 (insulin responsive sequence DNA binding protein-1)* and up-regulation of *calcium/calmodulin-dependent protein kinase II gamma*; *phospholipase A2, group VII*). Interestingly, in our present study, an adaptive and oxidative stress-responsive transcription factor [80-82], activating transcription factor 3 showed down-regulation in the heart in metabolic syndrome. Although, enhanced expression of activating transcription factor 3 has been reported to play a role in diabetic angiopathy [80], in stress-induced beta cell dysfunction [83,84] and hepatic LDL receptor down-regulation [85,86], its cardiac role in metabolic syndrome has not been implicated yet. Another stress inducible and regulator gene of eicosanoid biosynthesis, the phospholipase A2, group VII gene was up regulated in our present study in ZDF rat hearts. Increase of the expression of this gene was previously shown in ZDF rats in the liver and suggested to be a factor in the development of chronic low-grade inflammation in metabolic syndrome [87]. In our present study, a regulator gene of insulin action, the insulin responsive sequence DNA binding protein-1 showed down-regulation in metabolic syndrome in ZDF rat hearts. Down-regulation of this gene has been previously shown in the liver of diabetic [88] and obese [88] rats. However, it is unclear whether decreased expression of insulin responsive sequence DNA binding protein-1 is a consequence of insulin resistance or contributes to hyperglycemic phenotype. Calcium/calmodulin-dependent protein kinase II gamma showed up-regulation in ZDF hearts in our present study. This gene was reported to potentially mediate cardiac hypertrophy in pressure overload hypertension in mouse hearts [89].

In the present study, several genes related to the functional cluster of membrane proteins or receptors showed altered expression in ZDF hearts as compared to controls (e.g. down-regulation of *ATPase, (Na+)/K+ transporting, beta 4 polypeptide;* G protein-coupled receptor 37 and up-regulation of *ATPase, H+/K+ transporting, nongastric, alpha polypeptide; Huntingtin-associated protein 1).* Interestingly, here we have found gene expression changes of two members of the X,K-ATP-ase family due to metabolic syndrome. Surprisingly, ATPase, (Na+)/K+ transporting, beta 4 polypeptide showed down-regulation in obese ZDF rat hearts characterized by marked hyperlipidemia in the current study, however, this gene showed up-regulation in our previous study in cholesterol-induced hyperlidemia in the rat myocardium [77]. Additionally, it has been shown in the heart of spontaneously hypertensive rats that the microsomal Na+,K(+)-ATPase activity is reduced [90].

Another set of genes related to the functional cluster of structural proteins was found to be regulated differentially in hearts of ZDF rats as compared to controls (e.g. down-regulation of *myosin IXA and similar to collagen, type XXIV, alpha 1 (pred)* and up-regulation of *spectrin beta 3* and *aggrecan 1*). To our current knowledge, we are the first in the literature demonstrating cardiac gene expression changes of a novel epithelial extracellular matrix component [91], similar to collagen type XXIV; a cell migration regulator molecule [92], myosin IXA; a membrane stabilizer molecule [93], spectrin beta 3 and an extracellular matrix component proteoglycan [94], aggrecan 1, due to metabolic syndrome.

Some of the genes showing altered expression in ZDF rat hearts in the present study were not related to specific functional clusters or indicated as yet uncharacterized, predicted genes and fragments, the relevance of which should not be ignored. Many of these genes are reported for the first time in the literature to show altered expression in the heart due to metabolic syndrome including down-regulation e.g. of disintegrin-like and metallopeptidse (reprolysin type) with thrombospondin type 1 motif; G protein-coupled receptor 107 (predicted); S100 calcium binding protein A3; kallikrein 14 (predicted); neuronatin; connective tissue growth factor and up-regulation e.g. of amyloid beta (A4) precursor protein-binding, family A, member 1; similar to high density lipoprotein-binding protein (predicted); cationic trypsinogen; ubiquitin carboxy-terminal hydrolase L1.

In order to strengthen our results obtained by microarray analysis, and to provide some functional assessment, we have performed GO analysis on the genes showing altered expression due to metabolic syndrome. Significantly enriched GO terms were classified into three main categories including (i) cellular metabolic process, (ii) developmental process, and (iii) cellular localization. These results showed that metabolic syndrome may significantly affect several major biological processes, especially genes related to cellular metabolic processes and development (Table 5).

Our study is not without limitations. Our results regarding altered cardiac gene expression due to metabolic syndrome are based on determinations of approximately 15000 cardiac transcript levels, however, confirmation of gene expression changes at the protein level and direct measurement of the full rat transcriptome should be performed in the future. Moreover, additional studies providing more in-depth mechanistic insight and functional assessment should be carried out. Although our study does not specify which cell type (i.e. cardiomyocyte, fibroblast, smooth muscle cell, etc.) may be responsible for the observed alterations of cardiac gene expression due to metabolic syndrome, contribution of cardiomyocytes is likely the most significant [47,95].

In summary, we have found that 25 weeks old male ZDF rats develop severe metabolic syndrome and we have demonstrated for the first time that metabolic syndrome is associated with profound modifications of the cardiac transcriptome. Several of the genes showing altered expression in the hearts of ZDF rats have not been implicated in metabolic syndrome previously. We conclude that metabolic syndrome alters the gene expression pattern of the myocardium which may be involved in the development of cardiac pathologies in the state of metabolic syndrome. Based on our exploratory results, future studies should be carried out to investigate the precise role of specific genes in the development of cardiac consequences of metabolic syndrome to obtain deeper mechanistic insight.

### Grants

This work was supported by grants from the National Development Agency (MED_FOOD, Baross DA-TECH-07-2008-0041; TÁMOP-4.2.1/B-09/1/KONV-2010-0005; and TÁMOP-4.2.2/B-10/1-2010-0012), the Hungarian Scientific Research Fund (OTKA K79167), and co-financed by the European Regional Development Fund and VÁTI Hungarian Nonprofit Limited Liability Company for Regional Development and Town Planning (HURO/0901/137/2.2.2-HU-RO-TRANS-MED). T. Csont and A. Zvara hold a "János Bolyai Felowship" from the Hungarian Academy of Sciences.

## Competing interest

No conflicts of interest, financial or otherwise, are declared by the author(s).

## Authors’ contributions

Author contributions: L.G.P, .P.F., and T.C. conception and design of research; A.Z., V.F., N.G., G.F.K., C.C., J.P., and T.C. performed experiments; M.S., A.Z., V.F., N.G., G.S., C.C. and T.C.analyzed data; M.S., G.S., C.C., L.G.P., P.F., and T.C. interpreted results of experiments; M.S. and G.S. prepared figures; M.S., A.Z. and T.C. drafted manuscript; M.S., A.Z., T.C. and P.F., edited and revised manuscript; M.S., A.Z., V.F., N.G., G.S., J.P. L.G.P., C.C., G.F.K, P.F., and T.C. approved final version of manuscript.
